# Short-Term Exposure to Air Pollution and Cardiac Arrhythmia: A Meta-Analysis and Systematic Review

**DOI:** 10.3390/ijerph13070642

**Published:** 2016-06-28

**Authors:** Xuping Song, Yu Liu, Yuling Hu, Xiaoyan Zhao, Jinhui Tian, Guowu Ding, Shigong Wang

**Affiliations:** 1 Key Laboratory for Semi-Arid Climate Change of the Ministry of Education, College of Atmospheric Sciences, Lanzhou University, Lanzhou 730000, China; songxp15@lzu.edu.cn (X.S.); huyl10@lzu.edu.cn (Y.H.); zhaoxy15@lzu.edu.cn (X.Z.); 2 Center for Meteorological Environment and Human Health, Lanzhou University, Lanzhou 730000, China; 3 School of Public Health, Lanzhou University, Lanzhou 730000, China; yuliu14@lzu.edu.cn (Y.L.); dinggw@lzu.edu.cn (G.D.); 4 Evidence Based Medicine Center, School of Basic Medical Sciences, Lanzhou University, Lanzhou 730000, China; tjh996@163.com; 5 College of Atmospheric Sciences, Chengdu University of Information Technology, Chengdu 610225, China

**Keywords:** air pollution, cardiac arrhythmia, meta-analysis, systematic review

## Abstract

The objective was to assess the transient association between air pollution and cardiac arrhythmia. Five databases were searched for studies investigating the association between daily increases in air pollutants (PM_2.5_, PM_10_, carbon monoxide, nitrogen dioxide, sulfur dioxide and ozone) and arrhythmia hospitalization or arrhythmia mortality. Two reviewers independently selected studies, extracted data, and assessed risk of bias. Outcomes were analyzed via a random-effects model and reported as relative risk and 95% confidence interval. 25 studies satisfied our inclusion criteria and 23 contributed to the meta-analysis. Arrhythmia hospitalization or mortality were associated with increases in PM_2.5_ (RR = 1.015 per 10 μg/m^3^, 95% CI: 1.006–1.024), PM_10_ (RR = 1.009 per 10 μg/m^3^, 95% CI: 1.004–1.014), carbon monoxide (RR = 1.041 per 1 ppm, 95% CI: 1.017–1.065), nitrogen dioxide (RR = 1.036 per 10 ppb, 95% CI: 1.020–1.053), and sulfur dioxide (RR = 1.021 per 10 ppb, 95% CI: 1.003–1.039), but not ozone (RR = 1.012 per 10 ppb, 95% CI: 0.997–1.027). Both particulate and gaseous components, with the exception of ozone, have a temporal association with arrhythmia hospitalization or mortality. Compared with Europe and North America, a stronger association was noted in Asia.

## 1. Introduction

The adverse effects of air pollution on human health are of increasing concern throughout the world [1,2]. Air pollution is a complex mixture of particulate and gaseous components. The major particulate matter components are PM_2.5_ (fine particles) and PM_10_ (thoracic particles), which can be classified by aerodynamic diameter. Carbon monoxide (CO), nitrogen dioxide (NO_2_), sulfur dioxide (SO_2_) and ozone (O_3_) are the main gaseous pollutants. In recent years, numerous studies have researched the health effects of air pollution and cardiovascular disease (CVD) has caused extensive concern. Some large epidemiological and observational studies have shown that air pollution has adverse effects on cardiovascular health [3,4,5]. CVD is related to significant morbidity in industrialized countries [6]. Furthermore, it is the leading cause of death globally [7].

Cardiac arrhythmia, also called cardiac dysrhythmia, is defined as any change from the normal sequence of electrical impulses. In 2014, 2%–3% of the population in Europe and North America experienced atrial fibrillation [8]. About 50% of deaths due to CVD and 15% of all deaths are caused by sudden cardiac death, while approximately 80% of sudden cardiac deaths are due to ventricular arrhythmias [9]. Arrhythmia has distinct characteristics. It could lead to multiple complications. Therefore, identifying the risk factors and taking measurements are significant for decreasing burden of arrhythmia. The incidence of serious air pollution problems have increased in low-income and middle-income countries in recent years. Research has confirmed that economic development and pollution have an inverted-U relationship [10]. In other words, economic levels reflect the concentrations of air pollution to some degree. However, whether pollutant concentrations influence the threshold of adverse effects is unknown. Therefore, exploring the impact of pollutant concentrations on the relationship between air pollution and arrhythmia is a meaningful issue. In addition, studies conducted in different geographical locations have generally yielded inconsistent results. Therefore, the association on regional variations needs to be evaluated. The association between air pollution and arrhythmia remains controversial [11]. A series of studies have focused on the association between air pollution and specific types of CVD [12,13,14]. However, there is no systematic review and meta-analysis investigating the association in any language. Therefore, we conducted a comprehensive and systematic review to provide global evidence on the transient association between air pollution and cardiac arrhythmia.

## 2. Materials and Methods

The Preferred Reporting Items for Systematic Reviews and Meta-Analysis (PRISMA) were followed in the systematic review and meta-analysis [15]. The protocol was published online at the PROSPERO (registration number: CRD42016032635).

### 2.1. Databases

PubMed, Embase, Global Health, Cumulative Index to Nursing and Allied Health Literature (CINAHL) and Web of Science were systematically searched using the following terms: “arrhythmia”, “dysrhythmia”, “air pollution”, “particulate matter”, “ozone”, “carbon monoxide”, “nitrogen dioxide” and “sulfur dioxide”(Supplementary material Table S1). We limited our search to studies from inception to 20 June 2015, with no language restrictions. We also manually searched the reference lists of included studies and relevant reviews to identify remaining studies.

### 2.2. Selection of Articles and Extraction of Data

Case-crossover and time-series studies were included if they evaluated the short-term (up to seven days) association between air pollutants (gaseous pollutants: carbon monoxide, nitrogen dioxide, sulfur dioxide, or ozone; particulate components: PM_2.5_ or PM_10_) and arrhythmia hospitalization or mortality. However, studies were excluded if they collected arrhythmia data using implantable cardioverter defibrillator (ICD), home monitors and other equipments. In addition, studies investigating arrhythmia as comorbidity were also excluded. Arrhythmia consists of the following types: atrial fibrillation, bradycardia, conduction disorders, premature contraction, tachycardia, ventricular fibrillation and other rhythm disorders [16]. Studies investigating arrhythmia or specific types of arrhythmia were all included. Two investigators (Xuping Song and Yu Liu) independently retrieved titles and abstracts to identify eligible studies. Studies that could not be determined by titles and abstracts were selected by full-text screening. Any conflicts were adjudicated by a third investigator (Jinhui Tian). Authors were contacted for any uncertainty. Study characteristics were extracted using a standardized form including the following items: author, year of publication, location, period, study design, population age, outcome, air pollutants, number of events and data source.

### 2.3. Study Design

We included both case-crossover and time-series studies. The two techniques have their advantages and disadvantages. Case-crossover compares exposure in a case period when the event occurred with exposure in a specified control period. In this technique, cases serve as their own control, which can control stable subject-specific covariates, as well as seasonal patterns and secular trends using a time-stratified approach [17]. However, it assumes that time-varying risk factors are constant within reference periods. Therefore, case-crossover design is more useful in evaluating acute or transient effects [18]. Time-series design estimates the association between exposure and outcome using regression analysis. It could effectively control confounding factors. However, the selection used to control secular trends is not robust.

### 2.4. Data Synthesis

Relative risk (RR) was used as effect estimates in our study. It was converted to a standardized increment of pollutant concentration as follows: 10 μg/m^3^ for PM_2.5_ and PM_10_, 1 ppm for CO, 10 ppb for O_3_, NO_2_ and SO_2_. These levels have been commonly used in previous studies. The following formula was used to calculate the standardized risk estimates:

(1) $${\text{RR}}_{\left(\text{standardized}\right)}={\text{RR}}_{\left(\text{original}\right)}^{\text{Increment}\left(10\right)/\text{Increment}\left(\text{original}\right)}$$

Five studies did not offer overall risk, while stratified risk estimates by age [19], location [20] and temperature [21,22,23] were reported. In this case, the stratified estimates were pooled. Five studies [24,25,26,27,28] belonged to a subset, and the latest study [27] was included. One study [29] presented both results of case-crossover and time-series, and estimates of time-series were included in overall analysis.

### 2.5. Additional Analysis

To evaluate the heterogeneity, we conducted a stratified analysis on study design (case-crossover and time-series), age (all and older than 65 years), outcome (hospitalization and mortality), and geographical location (Europe, North America, and Asia). In addition, we pooled estimates of PM_2.5_ and PM_10_ stratified by pollutant concentrations as defined by the World Health Organization (WHO) air quality guidelines (AQG) [30]. Based on the distribution of particulate concentrations in the included studies, the AQG of 24-h concentrations and interim target-3 of annual mean concentrations were selected as stratification levels. The population attributable risks (PARs) on RRs of the overall analysis were calculated using the following formula [31]. PARs indicate the fraction of health outcome that can be attributed to exposure in a given population. In the formula, P_e_ represents the prevalence of air pollution exposure, and we assumed that P_e_ is 100%:

(2) $$\text{Population}-\text{attributable risks}=\frac{P_{\text{e}}\left(\text{RR}-1\right)}{P_{\text{e}}\left(\text{RR}-1\right)+1}$$

### 2.6. Quality Assessment

There is no standardized scale assessing the methodological quality of this type study. Therefore, we adopted a scale from related meta-analysis, which is composed of three items: validation of arrhythmia, exposure assessment, and adjustment of confounders [14].

#### 2.6.1. Validation of Arrhythmia

The diagnosis of arrhythmia is coded according to International Classification of Diseases (ICD)-9, or ICD-10, or is based on clinical and laboratory information.

#### 2.6.2. Exposure Assessment

The quality of pollutant measurement was assessed by measurement frequency. If it was conducted at least daily, we considered the study at low risk of bias. Otherwise, it was considered at high risk of bias.

#### 2.6.3. Adjustment of Confounders

Meteorological parameters, time trends, influenza epidemics, and seasonality are four items used to assess the adjustment of confounders. Studies adjusting at least three items were considered at low risk of bias.

### 2.7. Statistical Analysis

We anticipated significant heterogeneity between studies due to different study designs and locations. Therefore, random-effects model was performed. Heterogeneity between trials was assessed by the Chi‑square test and the extent of inconsistency was evaluated by the I^2^. Publication bias was assessed by Egger’s regression test [32]. To evaluate the heterogeneity, additional analyses were performed by study design (case-crossover and time-series), age (all and older than 65 years), outcome (hospitalization and mortality), and geographical location (Europe, North America, and Asia). In addition, the PARs on RRs of the overall analysis were calculated to assess the fraction of arrhythmia attributed to exposure. Most studies presented estimates for single lags (for example: lag 0, lag 1 and lag 2). Single lags were pooled separately where more than three estimates were available. The shortest lag was used to evaluate overall risk estimates. In addition, a few studies only presented cumulative lags, which were not suitable in the single lag analysis. However, the estimates of cumulative lags were used in the overall analysis. RR per standardized increment in pollutant concentration with 95% confidence intervals (CIs) was presented for statistic. Our analysis was performed with Stata software (Version 12.0, StataCorp, College Station, TX, USA). Statistical significance was taken as a two sided *p* < 0.05.

## 3. Results

We initially identified 546 studies, and 75 studies were reviewed in depth. Ultimately, 25 studies [19,20,21,22,23,27,29,33,34,35,36,37,38,39,40,41,42,43,44,45,46,47,48,49,50] met the inclusion criteria (Figure S1). Of these 25 studies, 11 used a case-crossover design, 13 used a time-series design, and one study used both study designs. Our meta-analysis incorporated two million events (Table S2, Table S3). We excluded two of the 25 studies in the quantity analysis, one study [34] did not present original data and the other [49] did not report 95% CIs of the effect estimates. Nineteen of the 25 studies reported hospitalization as an outcome, five reported mortality, and one study both reported hospitalization and mortality.

### 3.1. Overall Analysis

Both particulate and gaseous components, with the exception of ozone, have a temporal association with arrhythmia hospitalization or mortality (Figure S2). In addition, generally only the overall and lag 0 associations were significant for PM_2.5_, PM_10_, CO, NO_2_, and SO_2_. PM_2.5_ and PM_10_ both showed increase (RR = 1.015, 95% CI: 1.006–1.024) and (RR = 1.009, 95% CI: 1.004–1.014) respectively per 10 μg/m^3^ increment. In addition, PM_2.5_ and PM_10_ were only positively associated with arrhythmia hospitalization or mortality on the event day. CO presented the strongest association. The increase was (RR = 1.041, 95% CI: 1.017–1.065) per 1 ppm increment. NO_2_ (RR = 1.036, 95% CI: 1.020–1.053) and SO_2_ (RR = 1.021, 95% CI: 1.003–1.039) were also positively associated with arrhythmia hospitalization or mortality per 10 ppb increment. However, O_3_ presented no association (RR = 1.012 per 10 ppb, 95% CI: 0.997–1.027) per 10 ppb increment.

### 3.2. Subgroup Analysis

The subgroup analysis results by age, outcome, study design and geographical location were consistent with the overall analysis for each pollutant, with the exception of SO_2_ (Table 1). The subgroup analysis of age and outcome *showed* no relation between SO_2_ and arrhythmia hospitalization or mortality.

Daily concentrations of air pollutants by geographical location are shown in Table S4. We observed that the concentrations of PM_2.5_ and PM_10_ in Asia were nearly 2- to 3-fold higher than those in the other two studied continents and compared with Europe and North America, Asia showed a stronger association across all pollutants.

### 3.3. Stratification by Background Concentrations

Twelve studies [23,29,33,35,36,39,40,43,44,47,48,50] reported PM_10_. Studies ranking in the top three pollutant concentrations originated from developing countries (including China and Brazil). Pooled estimates showed that no association was found between PM_10_ and arrhythmia hospitalization or mortality (RR = 1.004 per 10 μg/m^3^, 95% CI: 0.992–1.017) in low concentrations (below 30 μg/m^3^). 

However, PM_10_ was positively associated with arrhythmia at increased levels (30–50 μg/m^3^: RR = 1.016 per 10 μg/m^3^, 95% CI: 1.004–1.027; >50 μg/m^3^: RR = 1.006 per 10 μg/m^3^, 95% CI: 1.004–1.007). A positive association was found between PM_2.5_ and arrhythmia hospitalization or mortality in all levels (Table 2).

### 3.4. Heterogeneity and Publication Bias

Heterogeneity, publication bias and PARs are presented in Table 3. The heterogeneity across all pollutants was most evident for NO_2_ (*I*^2^ = 92.0%) and least evident for SO_2_ (*I*^2^ = 72.3%). The results of Egger’s test illustrated that publication bias was noted for CO (*p* = 0.015).

## 4. Discussion

The systematic review and meta-analysis showed that PM_2.5_, PM_10_, CO, NO_2_, and SO_2_ could increase the risk for arrhythmia hospitalization or mortality. Nevertheless, ozone had no association. 

### 4.1. The Association between Air Pollution and Arrhythmia Maybe Stronger

Arrhythmia often shows no symptoms. Palpitations or feeling a pause between heartbeats are experienced in mild cases. Shortness of breath, light-headedness, syncope, or chest pain is reported in serious cases. Moreover, the majority of arrhythmia cases often lead to complications, such as heart failure, stroke or cardiac arrest [16]. Therefore, the number of hospitalizations and mortalities due to arrhythmia might be underestimated. Furthermore, data from monitoring stations might not represent personal exposure. The population density in the urban district is higher than that in the countryside. In addition, motor vehicle exhausts are an essential emission source contributing to air pollution [51]. Therefore, actual personal exposure might be higher.

### 4.2. Potential Mechanism between Arrhythmia and Air Pollution

The mechanism of the association between arrhythmia and air pollution has not been fully understood. There are several possible reasons for the association. The first hypothesis is autonomic nervous system dysfunction [52,53], which could lead to increased heart rate or decreased heart rate variability [54,55]. This mechanism was thought to be the most plausible reason for the association. The second hypothesis is that air pollution may affect blood coagulability. Particles can cross the pulmonary epithelium into the bloodstream [56,57]. The third hypothesis is inflammation [58]. Levels of inflammatory mediators would be higher due to air pollution [59].

### 4.3. Both Region and Pollutant Concentrations Affect Associations

Daily concentrations of PM_2.5_ and PM_10_ were higher in Asia, and both exceeded the recommend levels of WHO air quality guidelines (24-h concentrations: 25 μg/m^3^ and 50 μg/m^3^ respectively) [32]. Subgroup analysis on geographical location illustrated that increased risk of arrhythmia was found in Asia compared with Europe and North America. The additional analysis demonstrated that PM_10_ and arrhythmia are not associated at low pollutant concentrations. However, PM_10_ could elevate the risk of arrhythmia in increased concentrations. It indicates that the threshold for arrhythmia might be diverse in regions with varying pollutant concentrations. Research provides limited evidence on the threshold for adverse health effects. On the other hand, the thresholds for various types of diseases may be different. Therefore, the issue needs to be assessed further by more studies. Dose-response analyses and other type studies could provide evidence to identify the precise thresholds in different regions.

### 4.4. Implantable Cardioverter Defibrillator 

With increasing attention being paid to health outcomes, numerous studies are focused on the health effects of air pollution. Hospitalization and mortality due to disease are the most commonly used health outcomes. Our study applied arrhythmia hospitalization or mortality as well. In terms of arrhythmia, some patients are equipped with the ICD. Studies have proven that ICD is effective in preventing sudden cardiac death for high-risk patients [60,61]. In addition, ICD could continuously monitor arrhythmia and record the data and time of each episode. Therefore, many studies evaluated the relationship between arrhythmia detected by ICD and air pollution. Some have found a positive association between arrhythmia and air pollution, while others not [11,62,63,64,65,66]. This needs to be confirmed further via large sample studies.

### 4.5. Significant Points for Further Research

Firstly, more attention should be paid to the association between disease and the components of air pollution. For instance, PM has attracted increasing concern especially since the haze outbreak in Beijing, China. During 2004 to 2008, the 24-h mean concentration of PM_2.5_ was 105 μg/m^3^ in Beijing, which was up to 4.2-fold higher than the recommended level reported by WHO guidelines [67]. On 7 December 2015, Beijing issued a red alert for heavy air pollution for the first time [68]. Nowadays, a considerable number of studies investigate PM, while studies researching the specific components of PM (such as endotoxin, cell fragments, iron, copper, nickel, zinc and vanadium) are limited [58,69]. Moreover, it is significant to understand the mechanism between PM and diseases. Air pollution exposure assessments should also be improved. Outdoor concentrations could not assess personal exposure, particularly in cold seasons because people spend more time indoors [70,71].

### 4.6. Limitations

Potential limitations in our study need to be concerned. First, significant heterogeneities were observed in the analysis across all pollutants, possibly because data included in the meta-analysis came from five continents and several studies investigated the elderly population. Secondly, differences in study design and outcomes are also a potential source of heterogeneity. Thirdly, air pollution concentrations measured at monitoring sites likely caused misclassification bias for exposure, which is a common problem in environmental epidemiology. Finally, because most studies applied a single pollutant model instead of a multi-pollutant model, we assessed a single pollutant rather than multiple pollutants. To some extent, potential additive effects of multiple pollutants derived from the same emission source were ignored, such as motor vehicle exhausts and manufacturing.

### 4.7. Strengths

Firstly, a meta-analysis and systematically review investigating the association between air pollution and arrhythmia published in any language has not been performed. Five major databases were comprehensively searched. PM_2.5_, PM_10_, CO, NO_2_, SO_2_ and O_3_ were all analyzed in our study. Secondly, the analysis of geographical locations yielded contrasting results amongst Europe, North America, and Asia, which provided some crucial suggestions for global air pollution governance. Finally, we pooled estimates of PM_2.5_ and PM_10_ stratified by pollutant concentrations, which provided some evidence for identifying the arrhythmia thresholds.

## 5. Conclusions 

Both particulate and gaseous components, with the exception of ozone, have a temporal association with arrhythmia hospitalization or mortality. The strongest associations were observed at the same day of exposure across all pollutants. In addition, a stronger association was noted in Asia, compared with Europe and North America. Pollutant concentrations should be considered in assessing the health effects of air pollution. A vast quantity of studies on health effects of air pollution have been conducted in developed countries. However, air pollution is a global issue. High quality studies are required in developing countries, which could contribute to making effective measurements globally.

## Figures and Tables

**Table 1:** Subgroup analysis stratified by age, outcome, study design and geographical location across air pollutants.

Subgroup Analysis	PM_2.5_^§^			PM_10_			CO *		
	No. of Estimates	Relative Risk (95% CI)	*I*^2^^△^	No. of Estimates	Relative Risk (95% CI)	*I*^2^	No. of Estimates	Relative Risk (95% CI)	*I*^2^
Age									
All	15	1.018 (1.007, 1.028)	79.8%	9	1.010 (1.003, 1.017)	82.5%	8	1.060 (1.017, 1.104)	91.9%
≥65 years	3	1.009 (0.989, 1.028)	0	2	1.010 (1.002, 1.017)	0.0%	3	1.003 (0.982, 1.024)	0
Outcome									
Hospitalization	17	1.015 (1.005, 1.025)	78.4%	12	1.009 (1.004, 1.015)	79.7%	12	1.040 (1.017, 1.065)	90.9%
Mortality	3	1.027 (0.987, 1.068)	57.3%	2	1.009 (0.994, 1.024)	13.0%	3	1.077 (0.790, 1.467)	0
Study Design									
Case Crossover	13	1.017 (1.006, 1.028)	82.5%	6	1.012 (1.000, 1.024)	88.4%	6	1.076 (1.023, 1.133)	92.4%
Time Series	6	1.013 (1.003, 1.024)	0	8	1.006 (1.002, 1.011)	37.8%	9	1.026 (1.003, 1.050)	84.1%
Geographical Location									
Europe	2	0.997 (0.972, 1.023)	57.1%	3	1.003 (0.992, 1.014)	80.6%	3	1.002 (1.000, 1.004)	0
North America	12	1.007 (1.003, 1.011)	0	6	1.009 (0.999, 1.019)	55.4%	7	1.032 (1.002, 1.063)	78.8%
Asia	5	1.045 (1.011, 1.080)	85.2%	4	1.015 (1.003, 1.028)	88.8%	2	1.347 (1.236, 1.468)	30.8%
**Subgroup Analysis**	**O_3_^#^**			**NO_2_^¢^**			**SO_2_^¶^**		
	**No. of Estimates**	**Relative Risk (95% CI)**	***I*^2^**	**No. of Estimates**	**Relative Risk (95% CI)**	***I*^2^**	**No. of Estimates**	**Relative Risk (95% CI)**	***I*^2^**
Age									
All	8	1.019 (0.999, 1.039)	87.6%	9	1.037 (1.014, 1.061)	94.8%	9	1.018 (0.998, 1.037)	77.5%
≥65 years	2	0.993 (0.969, 1.017)	0	3	1.023 (1.003, 1.042)	0.0%	3	1.047 (0.985, 1.113)	33.5%
Outcome									
Hospitalization	9	1.016 (0.997, 1.035)	85.4%	13	1.036 (1.018, 1.055)	93.5%	10	1.020 (1.000, 1.040)	77.1%
Mortality	3	0.997 (0.975, 1.018)	55.4%	4	1.026 (1.003, 1.050)	23.6%	3	1.028 (0.988, 1.069)	0
Study Design									
Case Crossover	4	1.037 (0.997, 1.078)	94.1%	7	1.059 (1.027, 1.093)	91.4%	4	1.002 (0.989, 1.016)	0
Time Series	8	1.003 (0.996, 1.010)	0	10	1.021 (1.003, 1.040)	92.6%	9	1.022 (1.002, 1.042)	79.6%
Geographical Location									
Europe	3	1.002 (0.991, 1.014)	65.1%	4	1.016 (1.003, 1.030)	40.4%	3	1.010 (0.999, 1.021)	0
North America	6	0.998 (0.989, 1.008)	0	7	1.015 (1.003, 1.027)	66.3%	6	1.009 (0.992, 1.027)	32.4%
Asia	2	1.086 (1.033, 1.141)	69.6%	3	1.099 (1.044, 1.158)	90.6%	3	1.060 (1.043, 1.077)	0

**^§^** PM: Particulate Matter; * CO: Carbon Monoxide; **^#^** O_3_: Ozone; **^¢^** NO_2_: Nitrogen Dioxide; **^¶^** SO_2_: Sulfur Dioxide; **^△^** *I*^2^: Inconsistency Index.

**Table 2:** Association between particulate matter (PM_2.5_ and PM_10)_ and hospitalization or mortality due to arrhythmia stratified by pollutant concentrations.

Selected Levels	No. of Estimates	Relative Risk (95% CI *)	*I*^2^^△^
PM_2.5_^¶^			
～15 μg/m^3^	12	1.005 (1.001, 1.009)	0
15 μg/m^3^–25 μg/m^3^	3	1.019 (1.006, 1.033)	0
25 μg/m^3^～	4	1.053 (1.012, 1.094)	87.4%
PM_10_			
～30 μg/m^3^	5	1.004 (0.992, 1.017)	69.6%
30 μg/m^3^–50 μg/m^3^	6	1.016 (1.004, 1.027)	84.0%
50 μg/m^3^～	2	1.006 (1.004, 1.007)	0

**^¶^** PM: Particulate Matter; **^△^** *I*^2^: Inconsistency Index.

**Table 3:** Heterogeneity, publication bias and population attributable risks across air pollutants.

Item	PM_2.5_ (μg/m^3^) ^¶^	PM_10_ (μg/m^3^)	CO (ppm)	Ozone (ppb)	NO_2_ (ppb)	SO_2_ (ppb)
Increment	10	10	1	10	10	10
No. of estimates	19	13	14	11	16	12
No. of studies	11	12	12	10	14	11
Pollutant concentration:						
Median	15.272	30.737	1.021	23.793	24.885	4.038
Q1–Q3 **^#^**	9.860–21.425	21.530–48.222	0.702–1.438	15.976–33.165	18.464–31.951	2.333–9.257
Heterogeneity	75.70%	77.80%	89.40%	82.60%	92%	72.30%
RR (95% CI) *	1.015 (1.006, 1.024)	1.009 (1.004, 1.014)	1.041 (1.017, 1.065)	1.012 (0.997, 1.027)	1.036 (1.020, 1.053)	1.021 (1.003, 1.039)
*p* value **^§^**	0.001	0.001	0.001	0.115	<0.001	0.025
Egger regression test (*p* value)	0.132	0.365	0.015	0.305	0.726	0.555
PARs (95% CI) **^¢^**	0.015 (0.006, 0.023)	0.009 (0.004, 0.014)	0.039 (0.017, 0.061)	0.012 (−0.003, 0.026)	0.035 (0.020, 0.050)	0.021 (0.003, 0.038)

* Risk estimates of overall analysis across air pollutants. **^§^** *p* value: *p* value of overall analysis across air pollutants. **^¢^** PARs (population attributable risks) = P_e_ (RR − 1)/(P_e_(RR − 1) + 1).**^¶^** PM: Particulate Matter. **^#^** Q1: first quartile value. Q3: third quartile value. ppm: parts per million. ppb: parts per billion.

## Supplementary Materials

The following are available online at www.mdpi.com/1660-4601/13/7/642/s1, Table S1: Search strategy for PubMed, Table S2: Details of included studies in the systematic review, Table S3: Characteristics and quality assessment of included studies in the systematic review, Table S4: Daily concentrations of air pollutants by geographical location, Figure S1: Flow chart of the literature screening process, Figure S2: Association between particulate and gaseous components with hospitalization or mortality due to arrhythmia.

## References

[1] Brunekreef B., Holgate S.T. (2002). Air pollution and health. Lancet.

[2] Kampa M., Castanas E. (2008). Human health effects of air pollution. Environ. Pollut.

[3] Hoek G., Brunekreef B., Goldbohm S., Fischer P., van den Brandt P.A. (2002). Association between mortality and indicators of traffic-related air pollution in the netherlands: A cohort study. Lancet.

[4] Miller K.A., Siscovick D.S., Sheppard L., Shepherd K., Sullivan J.H., Anderson G.L., Kaufman J.D. (2007). Long-term exposure to air pollution and incidence of cardiovascular events in women. N Engl J. Med..

[5] Pope C.A., Burnett R.T., Thurston G.D., Thun M.J., Calle E.E., Krewski D., Godleski J.J. (2004). Cardiovascular mortality and long-term exposure to particulate air pollution: Epidemiological evidence of general pathophysiological pathways of disease. Circulation.

[6] Ayres J. (2011). Cardiovascular Disease and Air Pollution: A Report by the Committee on the Medical Effects of Air Pollutants. https://www.gov.uk/government/publications/comeap-cardiovascular-disease-and-air-pollution.

[7] Bhaskaran K., Hajat S., Haines A., Herrett E., Wilkinson P., Smeeth L. (2009). Effects of air pollution on the incidence of myocardial infarction. Heart.

[8] Zoni-Berisso M., Lercari F., Carazza T., Domenicucci S. (2014). Epidemiology of atrial fibrillation: European perspective. Clin. Epidemiol..

[9] Mehra R. (2007). Global public health problem of sudden cardiac death. J. Electrocardiol..

[10] Selden T.M., Song D. (1994). Environmental quality and development: Is there a kuznets curve for air pollution emissions?. J. Environ. Econ. Manag..

[11] Dockery D.W., Luttmann-Gibson H., Rich D.Q., Link M.S., Mittleman M.A., Gold D.R., Koutrakis P., Schwartz J.D., Verrier R.L. (2005). Association of air pollution with increased incidence of ventricular tachyarrhythmias recorded by implanted cardioverter defibrillators. Environ. Health Perspect.

[12] Mustafic H., Jabre P., Caussin C., Murad M.H., Escolano S., Tafflet M., Perier M.C., Marijon E., Vernerey D., Empana J.P. (2012). Main air pollutants and myocardial infarction: A systematic review and meta-analysis. JAMA.

[13] Shah A.S., Langrish J.P., Nair H., McAllister D.A., Hunter A.L., Donaldson K., Newby D.E., Mills N.L. (2013). Global association of air pollution and heart failure: A systematic review and meta-analysis. Lancet.

[14] Shah A.S., Lee K.K., McAllister D.A., Hunter A., Nair H., Whiteley W., Langrish J.P., Newby D.E., Mills N.L. (2015). Short term exposure to air pollution and stroke: Systematic review and meta-analysis. BMJ.

[15] Moher D., Liberati A., Tetzlaff J., Altman D.G. (2009). Preferred reporting items for systematic reviews and meta-analyses: The prisma statement. Ann. Intern. Med..

[16] American Heart Association About Arrhythmia. http://www.heart.org/HEARTORG/Conditions/Arrhythmia/AboutArrhythmia/About-Arrhythmia_UCM_002010_Article.jsp#main.

[17] Fung K.Y., Krewski D., Chen Y., Burnett R., Cakmak S. (2003). Comparison of time series and case-crossover analyses of air pollution and hospital admission data. Int. J. Epidemiol..

[18] Lumley T., Levy D. (2000). Bias in the case-crossover design: Implications for studies of air pollution. Environmetrics.

[19] Barnett A.G., Williams G.M., Schwartz J., Best T.L., Neller A.H., Petroeschevsky A.L., Simpson R.W. (2006). The effects of air pollution on hospitalizations for cardiovascular disease in elderly people in Australian and New Zealand cities. Environ. Health Perspect.

[20] Talbott E.O., Rager J.R., Benson S., Brink L.A., Bilonick R.A., Wu C. (2014). A case-crossover analysis of the impact of PM_2.5_ on cardiovascular disease hospitalizations for selected cdc tracking states. Environ. Res..

[21] Chang C.C., Chen P.S., Yang C.Y. (2015). Short-term effects of fine particulate air pollution on hospital admissions for cardiovascular diseases: A case-crossover study in a tropical city. J. Toxicol. Environ. Health A.

[22] Chiu H.F., Tsai S.S., Weng H.H., Yang C.Y. (2013). Short-term effects of fine particulate air pollution on emergency room visits for cardiac arrhythmias: A case-crossover study in taipei. J. Toxicol. Environ. Health A.

[23] Tsai S.S., Chiu H.F., Wu T.N., Yang C.Y. (2009). Air pollution and emergency room visits for cardiac arrhythmia in a subtropical city: Taipei, taiwan. Inhal. Toxicol..

[24] Goldberg M.S., Bailar J.C., Burnett R.T., Brook J.R., Tamblyn R., Bonvalot Y., Ernst P., Flegel K.M., Singh R.K., Valois M.F. (2000). Identifying subgroups of the general population that may be susceptible to short-term increases in particulate air pollution: A time-series study in montreal, quebec. Res. Rep. Health Eff. Inst..

[25] Goldberg M., Burnett R., Bailar J., Brook J., Bonvalot Y., Tamblyn R., Singh R., Valois M. (2001). The association between daily mortality and short-term effects of ambient air particle pollution in Montreal, Quebec: 1. Nonaccidental mortality. Environ. Res. A.

[26] Goldberg M.S., Burnett R., Bailar J.C., Brook J., Bonvalot Y., Tamblyn R., Singh R., Valois M.F., Vincent R. (2001). The association between daily mortality and short-term effects of ambient air particle pollution in Montreal, Quebec: 2. Cause-specific mortality. Environ. Res. C.

[27] Goldberg M.S., Burnett R.T., Stieb D.M., Brophy J.M., Daskalopoulou S.S., Valois M.F., Brook J.R. (2013). Associations between ambient air pollution and daily mortality among elderly persons in Montreal, Quebec. Sci. Total Environ..

[28] Goldberg M., Burnett R. (2003). Revised Analyses of Time-Series Studies of Air Pollution and Health: Special Report.

[29] Peel J.L., Metzger K.B., Klein M., Flanders W.D., Mulholland J.A., Tolbert P.E. (2007). Ambient air pollution and cardiovascular emergency department visits in potentially sensitive groups. Am. J. Epidemiol..

[30] World Health Organization WHO Air Quality Guidelines for Particulate Matter, Ozone, Nitrogen Dioxide and Sulfur Dioxide. http://otp.unesco-ci.org/zh-hans/node/3671.

[31] Northridge M.E. (1995). Public health methods-attributable risk as a link between causality and public health action. Am. J. Public Health.

[32] Egger M., Davey Smith G., Schneider M., Minder C. (1997). Bias in meta-analysis detected by a simple, graphical test. BMJ.

[33] Guo Y.M., Liu L.Q., Chen J.M., Yang M.J., Wich M., Pan X.C. (2008). Association between sulfur dioxide air pollution and hospital emergency treatment for circulatory diseases: A case-crossover study. J. Environ. Health.

[34] Bunch T.J., Horne B.D., Asirvatham S.J., Day J.D., Crandall B.G., Weiss J.P., Osborn J.S., Anderson J.L., Muhlestein J.B., Lappe D.L. (2011). Atrial fibrillation hospitalization is not increased with short-term elevations in exposure to fine particulate air pollution. Pacing Clin. Electrophysiol..

[35] Zhao A., Chen R., Kuang X., Kan H. (2014). Ambient air pollution and daily outpatient visits for cardiac arrhythmia in Shanghai, China. J. Epidemiol..

[36] Santos U.P., Terra-Filho M., Lin C.A., Pereira L.A., Vieira T.C., Saldiva P.H., Braga A.L. (2008). Cardiac arrhythmia emergency room visits and environmental air pollution in Sao Paulo, Brazil. J. Epidemiol. Community Health.

[37] Ueda K., Nitta H., Ono M. (2009). Effects of fine particulate matter on daily mortality for specific heart diseases in Japan. Circ. J..

[38] Poloniecki J.D., Atkinson R.W., de Leon A.P., Anderson H.R. (1997). Daily time series for cardiovascular hospital admissions and previous day’s air pollution in Londozn, UK. Occup. Environ. Med..

[39] Hoek G., Brunekreef B., Verhoeff A., Wijnen J.W., Fischer P. (2000). Daily mortality and air pollution in the netherlands. J. Air Waste Manag. Assoc..

[40] Burnett R.T., Smith-Doiron M., Stieb D., Cakmak S., Brook J.R. (1999). Effects of particulate and gaseous air pollution on cardiorespiratory hospitalizations. Arch. Environ. Health.

[41] Halonen J.I., Lanki T., Yli-Tuomi T., Tiittanen P., Kulmala M., Pekkanen J. (2009). Particulate air pollution and acute cardiorespiratory hospital admissions and mortality among the elderly. Epidemiology.

[42] Koken P.J., Piver W.T., Ye F., Elixhauser A., Olsen L.M., Portier C.J. (2003). Temperature, air pollution, and hospitalization for cardiovascular diseases among elderly people in Denver. Environ. Health Perspect..

[43] Linn W.S., Szlachcic Y., Gong H., Kinney P.L., Berhane K.T. (2000). Air pollution and daily hospital admissions in metropolitan Los Angeles. Environ. Health Perspect..

[44] Stieb D.M., Szyszkowicz M., Rowe B.H., Leech J.A. (2009). Air pollution and emergency department visits for cardiac and respiratory conditions: A multi-city time-series analysis. Environ. Health.

[45] Haley V.B., Talbot T.O., Felton H.D. (2009). Surveillance of the short-term impact of fine particle air pollution on cardiovascular disease hospitalizations in New York state. Environ. Health.

[46] Chiusolo M., Cadum E., Stafoggia M., Galassi C., Berti G., Faustini A., Bisanti L., Vigotti M.A., Dessi M.P., Cernigliaro A. (2011). Short-term effects of nitrogen dioxide on mortality and susceptibility factors in 10 Italian cities: The EPIAIR study. Environ. Health Perspect..

[47] Colais P., Faustini A., Stafoggia M., Berti G., Bisanti L., Cadum E., Cernigliaro A., Mallone S., Pacelli B., Serinelli M. (2012). Particulate air pollution and hospital admissions for cardiac diseases in potentially sensitive subgroups. Epidemiology.

[48] Milojevic A., Wilkinson P., Armstrong B., Bhaskaran K., Smeeth L., Hajat S. (2014). Short-term effects of air pollution on a range of cardiovascular events in England and Wales: Case-crossover analysis of the MINAP database, hospital admissions and mortality. Heart.

[49] Tolbert P.E., Klein M., Metzger K.B., Peel J., Flanders W.D., Todd K., Mulholland J.A., Ryan P.B., Frumkin H. (2000). Interim results of the study of particulates and health in Atlanta (Sophia). J. Expo. Anal. Environ. Epidemiol..

[50] Lippmann M., Ito K., Nadas A., Burnett R.T. (2000). Association of particulate matter components with daily mortality and morbidity in urban populations. Res. Rep. Health Eff. Inst..

[51] Mills N.L., Donaldson K., Hadoke P.W., Boon N.A., MacNee W., Cassee F.R., Sandstrom T., Blomberg A., Newby D.E. (2009). Adverse cardiovascular effects of air pollution. Nat. Clin. Pract. Cardiovasc. Med..

[52] Rodney H., Falk M.D. (2001). Medical progress: Atrial fibrillation. N. Engl. J. Med..

[53] Kennedy H.L. (1997). Beta blockade, ventricular arrhythmias, and sudden cardiac death. Am. J. Cardiol.

[54] Pope C.A., Verrier R.L., Lovett E.G., Larson A.C., Raizenne M.E., Kanner R.E., Schwartz J., Villegas G.M., Gold D.R., Dockery D.W. (1999). Heart rate variability associated with particulate air pollution. Am. Heart J..

[55] Pope C.A., Hansen M.L., Long R.W., Nielsen K.R., Eatough N.L., Wilson W.E., Eatough D.J. (2004). Ambient particulate air pollution, heart rate variability, and blood markers of inflammation in a panel of elderly subjects. Environ. Health Perspect..

[56] Lucking A.J., Lundback M., Mills N.L., Faratian D., Barath S.L., Pourazar J., Cassee F.R., Donaldson K., Boon N.A., Badimon J.J. (2008). Diesel exhaust inhalation increases thrombus formation in man. Eur. Heart J..

[57] Nemmar A., Hoylaerts M.F., Hoet P.H., Nemery B. (2004). Possible mechanisms of the cardiovascular effects of inhaled particles: Systemic translocation and prothrombotic effects. Toxicol. Lett..

[58] Brook R.D., Franklin B., Cascio W., Hong Y., Howard G., Lipsett M., Luepker R., Mittleman M., Samet J., Smith S.C. (2004). Air pollution and cardiovascular disease: A statement for healthcare professionals from the expert panel on population and prevention science of the american heart association. Circulation.

[59] Brauner E.V., Moller P., Barregard L., Dragsted L.O., Glasius M., Wahlin P., Vinzents P., Raaschou-Nielsen O., Loft S. (2008). Exposure to ambient concentrations of particulate air pollution does not influence vascular function or inflammatory pathways in young healthy individuals. Part. Fibre Toxicol..

[60] Buxton A.E., Lee K.L., Fisher J.D., Josephson M.E., Prystowsky E.N., Hafley G. (1999). A randomized study of the prevention of sudden death in patients with coronary artery disease: Multicenter unsustained tachycardia trial investigators. N. Engl. J. Med..

[61] Moss A.J., Hall W.J., Cannom D.S., Daubert J.P., Higgins S.L., Klein H., Levine J.H., Saksena S., Waldo A.L., Wilber D. (1996). Improved survival with an implanted defibrillator in patients with coronary disease at high risk for ventricular arrhythmia. Multicenter automatic defibrillator implantation trial investigators. N. Engl. J. Med..

[62] Dockery D.W., Luttmann-Gibson H., Rich D.Q., Link M.S., Schwartz J.D., Gold D.R., Koutrakis P., Verrier R.L., Mittleman M.A. (2005). Particulate air pollution and nonfatal cardiac events—Part II: Association of air pollution with confirmed arrhythmias recorded by implanted defibrillators. Res. Rep. Health Eff. Inst..

[63] Peters A., Liu E., Verrier R.L., Schwartz J., Gold D.R., Mittleman M., Baliff J., Oh J.A., Allen G., Monahan K. (2000). Air pollution and incidence of cardiac arrhythmia. Epidemiology.

[64] Rich D., Schwartz J., Mittleman M., Link M., Luttmann-Gibson H., Catalano P. (2005). Association of ambient air pollution and ICD-detected ventricular arrhythmias in Boston, MA. Am. J. Epidemiol..

[65] Rich K.E., Petkau J., Vedal S., Brauer M. (2004). A case-crossover analysis of particulate air pollution and cardiac arrhythmia in patients with implantable cardioverter defibrillators. Inhal. Toxicol..

[66] Vedal S., Rich K., Brauer M., White R., Petkau J. (2004). Air pollution and cardiac arrhythmias in patients with implantable cardioverter defibrillators. Inhal. Toxicol..

[67] Guo Y., Li S., Tian Z., Pan X., Zhang J., Williams G. (2013). The burden of air pollution on years of life lost in Beijing, China, 2004–2008: Retrospective regression analysis of daily deaths. BMJ.

[68] Many Cities Battle Against Haze in China and Beijing Issued Red Alert for First Time. http://finance.sina.cn/2015–12–07/detail-ifxmhqac0153460.d.html?from=baidu&stun=20007&vt=4.

[69] Sun X., Luo X., Zhao C., Zhang B., Tao J., Yang Z., Ma W., Liu T. (2015). The associations between birth weight and exposure to fine particulate matter (PM) and its chemical constituents during pregnancy: A meta-analysis. Environ. Pollut..

[70] Sarnat J.A., Schwartz J., Catalano P.J., Suh H.H. (2001). Gaseous pollutants in particulate matter epidemiology: Confounders or surrogates?. Environ. Health Perspect..

[71] Sarnat J.A., Brown K.W., Schwartz J., Coull B.A., Koutrakis P. (2005). Ambient gas concentrations and personal particulate matter exposures: Implications for studying the health effects of particles. Epidemiology.

