# Supplementary material for: Short-Term Exposure to Air Pollution and Cardiac Arrhythmia: A Meta-Analysis and Systematic Review
Source: Int J Environ Res Public Health. 2016 Jun 28;13(7):642. doi: 10.3390/ijerph13070642 (PMC4962183; doi:10.3390/ijerph13070642)
Supplement: Supplementary file 1 [file ijerph-13-00642-s001.pdf]

Supplementary Materials: Short-Term Exposure to Air Pollution and Cardiac Arrhythmia: A Meta-Analysis and Systematic Review

Xuping Song, Yu Liu, Yuling Hu, Xiaoyan Zhao, Jinhui Tian, Guowu Ding and Shigong Wang

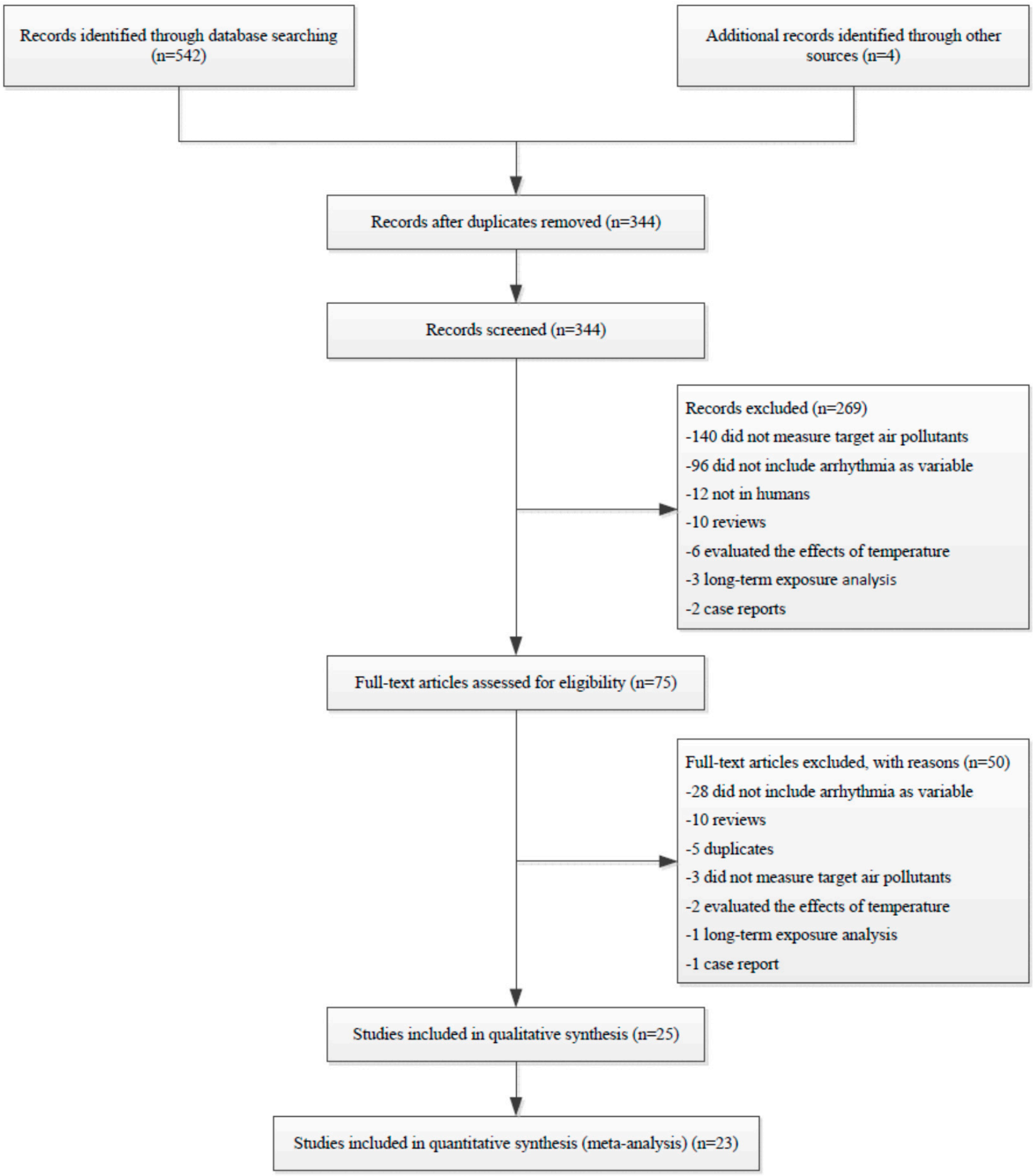

Figure S1. Flow chart of the literature screening process.

	No. of Events	No. of Estimates	Relative Risks (95%CI)
**$PM_{2.5}$ (per 10 μg/m^3 increment)**			
Overall	1 642 094	20	1.015 (1.006, 1.024)
lag0d	1 229 406	15	1.010 (1.003, 1.016)
lag1d	1 212 685	13	1.002 (0.998, 1.005)
lag2d	1 212 685	13	0.997 (0.994, 1.001)
lag3d	128 226	3	0.995 (0.990, 1.000)
**PM_{10} (per 10 μg/m^3 increment)**			
Overall	684 904	13	1.009 (1.004, 1.014)
lag0d	241 727	9	1.011 (1.004, 1.017)
lag1d	110 921	4	0.999 (0.997, 1.000)
lag2d	110 921	4	1.000 (0.998, 1.001)
**CO (per 1 ppm increment)**			
Overall	723 570	14	1.041 (1.017, 1.065)
lag0d	174 029	7	1.108 (1.034, 1.187)
lag1d	106 575	4	1.004 (0.958, 1.052)
lag2d	76 147	3	1.011 (0.894, 1.144)
**O_3 (per 10 ppb increment)**			
Overall	644 383	11	1.012 (0.997, 1.027)
lag0d	170 778	6	1.023 (0.988, 1.059)
lag1d	106 575	4	1.006 (0.993, 1.019)
lag2d	76 147	3	0.991 (0.971, 1.011)
**NO_2 (per 10 ppb increment)**			
Overall	815 039	16	1.036 (1.020, 1.053)
lag0d	230 969	8	1.047 (1.019, 1.075)
lag1d	163 515	5	1.010 (0.997, 1.023)
lag2d	133 087	4	1.005 (0.996, 1.013)
**SO_2 (per 10 ppb increment)**			
Overall	629 482	12	1.021 (1.003, 1.039)
lag0d	155 877	7	1.037 (1.002, 1.073)
lag1d	163 515	5	1.012 (1.003, 1.021)
lag2d	133 087	4	0.996 (0.966, 1.028)

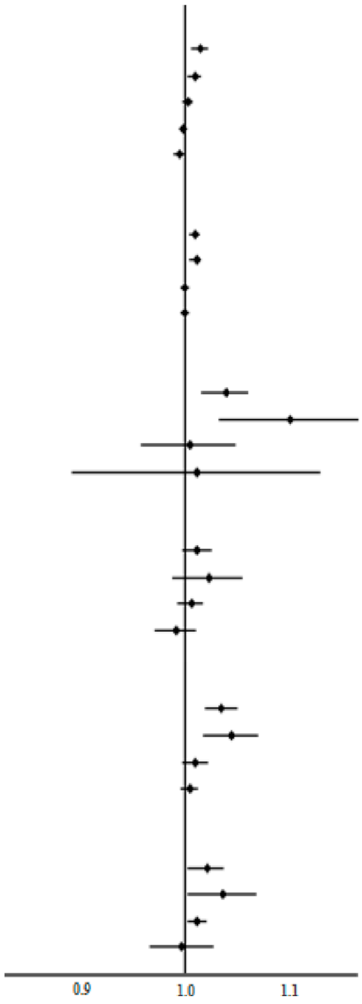

Figure S2. Association between particulate and gaseous components with hospitalization or mortality due to arrhythmia.

Table S1. Search Strategy for PubMed.

No.	Search Strategy
#1	air pollution*/or air pollutant*/or air polluted/or air contamination*/or atmosphere pollution*/or atmosphere pollutant*/ or atmosphere contamination*/or atmospheric pollution*/or atmospheric pollutant*/or atmospheric contamination*/or “particulate matter”/or “PM_{10}”/or “$PM_{2.5}$”/or ozone/ or “O_3”/or “carbon monoxide”/or carbonmonoxide/or “CO“/or “nitrogen dioxide”/or “NO_2”/or “sulphur dioxide”/or “sulphur dioxyde”/or “sulfurous anhydride“/or “SO_2”.ti,ab.
#2	Air Pollution/or Particulate Matter/or Ozone/or Carbon Monoxide/or Nitrogen Dioxide/or Sulfur Dioxide.sh.
#3	or/1,2
#4	arrhythmia* /or dysrhythmia* /or “CA”.ti,ab.
#5	Arrhythmias, Cardiac.sh.
#6	“Sick Sinus Syndrome”/or “SSS”/or “Sick Sinus Node Syndrom”/or Sinus Node Dysfunction*/or Sinus Node Disease*/or Sinus Arrest*.ti,ab.
#7	Arrhythmia, Sinus/or Sick Sinus Syndrome/or Sinus Arrest, Cardiac.sh.
#8	atrial fibrillation*/or auricular fibrillation*/or “AF".ti,ab.
#9	Atrial Fibrillation.sh.
#10	Atrial Flutter*/or Auricular Flutter*.ti,ab.
#11	Atrial Flutter.sh.
#12	Bradycardia*/or Bradyarrhythmia*.ti,ab.
#13	Bradycardia.sh.
#14	“St Segment Elevation“/or “Sudden Death Syndrome“/or “Sudden Unexplained Death Syndrome“/or “Brugada ECG Pattern“.ti,ab.
#15	Brugada Syndrome.sh.
#16	Premature Beat*/or Extrasystole*/or Ectopic Heartbeat*/or Atrial Premature Complex*/or Premature Supraventricular Beat*/or Premature Atrial Beat*/or “Premature Atrial Complex“/or Premature Atrial Contraction*/or Atrial Ectopic Beat*/or Atrial Extrasystole*/or Premature Ventricular Beat*/or "Premature Ventricular Complex“/or Ventricular Ectopic Beat*/or “Ventricular Premature Complex“/or Ventricular Extrasystole*/or Premature Ventricular Contraction*.ti,ab.
#17	Cardiac Complexes, Premature/or Atrial Premature Complexes/or Ventricular Premature Complexes.sh.
#18	"Commotio Cordis“/or Cardiac Concussion*.ti,ab.
#19	Commotio Cordis.sh.
#20	Heart Block*/or Auriculo-Ventricular Dissociation*/or Atrioventricular Dissociation*/or A V Dissociation*/or “Adams Stokes Syndrome"/or “Stokes Adams Attacks“/or “Stokes Adams Syndrome“/or Atrioventricular Block*/or AV Block*/or Atrioventricular Conduction Block*/or Bundle Branch Block*/or Fascicular Block*/or Sinoatrial Block*/or Sinoatrial Exit Block*.ti,ab.
#21	Heart Block/or Adams-Stokes Syndrome/or Atrioventricular Block/or Bundle-Branch Block/or Sinoatrial Block.sh.
#22	“Andersen Syndrome“/or “Andersen Tawil Syndrome“/or “Jervell Lange Nielsen Syndrome“/or Surdo Cardiac Syndrome*/or “Romano Ward Syndrome“/or “Long QT Syndrome“.ti,ab.
#23	Long QT Syndrome/or Andersen Syndrome/or Jervell-Lange Nielsen Syndrome/or Romano-Ward Syndrome.sh.
#24	Parasystole*.ti,ab.
#25	Parasystole.sh.
#26	Preexcitation Syndrome*/or Pre Excitation Syndrome*/or “Lown Ganong Levine Syndrome“/or “Parkinson White Syndrome“/or “WPW Syndrome“.ti,ab.
#27	Pre-Excitation Syndromes/or Lown-Ganong-Levine Syndrome/or Pre-Excitation, Mahaim-Type/or Wolff-Parkinson-White Syndrome.sh.
#28	Tachyarrhythmia*/or Tachycardia*/or Accelerated Idioventricular Rhythm*/or “AIVR“/or “Torsade de Pointes"/or “Torsades de Pointes“.ti,ab.

Table S1. *Cont.*

No.	Search Strategy
#29	Tachycardia/or Tachycardia, Paroxysmal/or Tachycardia, Reciprocating/or Tachycardia, Atrioventricular Nodal Reentry/or Tachycardia, Sinoatrial Nodal Reentry/or Tachycardia, Supraventricular/or Tachycardia, Ectopic Atrial/or Tachycardia, Ectopic Junctional/or Tachycardia, Sinus/or Tachycardia, Ventricular/or Accelerated Idioventricular Rhythm/or Torsades de Pointes.sh.
#30	Ventricular Fibrillation*.ti,ab.
#31	Ventricular Fibrillation.sh.
#32	Ventricular Flutter*.ti,ab.
#33	Ventricular Flutter.sh.
#34	or/3-33
#35	"time series".all.
#36	"case crossover".all.
#37	or/35,36
#38	and/3,34,37

Table S2. Details of included studies in the systematic review.

Author	Year	Location	Period	Study Design	Outcome	Population	No. of Events	Data Source
Barnett et al.	2006	Australia and New Zealand	1998–2001	Case-Crossover	HA §	≥15 years	NR *	Government health departments (Australia) and ministry of health (New Zealand)
Talbott et al.	2014	USA	2001–2008	Case-Crossover	HA	All	1,008,901	Hospital discharge records
Guo et al.	2008	China	2004–2006	Case-Crossover	HA	All	1149	Emergency department registry
Chiu et al.	2013	Taiwan	2006–2010	Case-Crossover	HA	All	16,721	Medicare data
Tsai et al.	2009	Taiwan	2000–2006	Case-Crossover	HA	All	21,581	Medicare data
Bunch et al.	2011	USA	1993–2008	Case-Crossover	HA	All	10,457	Medicare data
Zhao et al.	2014	China	2010–2011	Time-Series	HA	All	56,940	Hospital outpatient records
Santos et al.	2008	Brazil	1998–1999	Time-Series	HA	≥17 years	3251	Heart institute of the university of sao paulo medical school
Ueda et al.	2009	Japan	2002–2004	Time-Series	Mortality	All	7083	Ministry of health
Poloniecki et al.	1997	UK	1987–1994	Time-Series	HA	All	NR	Hospital episode records
Hoek et al.	2011	Netherland	1986–1994	Time-Series	Mortality	All	NR	Death certificates central beareu of statistics
Burnett et al.	1999	Canada	1980–1994	Time-Series	HA	All	NR	Ontario Ministry of health
Halonen et al.	2009	Finland	1998–2004	Time-Series	Mortality	≥65 years	10,423	National registers
Koken et al.	2003	USA	1993–1997	Time-Series	HA	>65 years	NR	Agency for healthcare research and quality
Linn et al.	2000	USA	1992–1995	Time-Series	HA	≥30 years	NR	Office of statewide health planning and development (OSHPD)
Stieb et al.	2009	Canada	1992–2003	Time-Series	HA	All	45,160	Emergency department registry
Haley et al.	2009	USA	2001–2005	Case-Crossover	HA	≥35 years	110,131	New York state hospitals and deparment of health registry
Chiusolo et al.	2011	Italy	2001–2005	Case-Crossover	Mortality	>35 years	34,529	Census office registry
Peel et al.	2007	USA	1993–2000	Both	HA	All	12,839	Emergency department billing data
Colais et al.	2012	Italy	2001–2005	Case-Crossover	HA	≥65 years	32,924	Hospital discharge registry
Chang et al.	2015	Taiwan	2006–2010	Case-Crossover	HA	All	5688	National health insurance (NHI)
Milojevic al.	2014	UK	2003–2009	Case-Crossover	Both	All	391,308	MINAP ¢, HES §§, Mortality (office for national statistics)
Tolbert et al.	2000	USA	1993–2000	Time-Series	HA	All	4099	Billing data
Goldberg et al.	2013	Canada	1990–2003	Time-Series	Mortality	≥65 years	23,315	Death certificates
Lippmann et al.	2000	Canada	1992–1994	Time-Series	HA	≥65 years	7672	Medicare data

* NR: Not Reported.; § HA: Hospital Admissions. ¢ MINAP: The Myocardial Ischaemia National Audit Project. §§ HES: Hospital Episode Statistics. Bunch et al. and Tolbert et al. did not presented complete data and were excluded in meta-analysis.

Table S3. Characteristics and quality assessment of included studies in the systematic review.

Author	Year	Single/Multi City	Single Lag	Shortest Lag *	Diagnostic Evidence of Disease	Daily Pollutant Measurement	Season	Time Trends	Meteorological Parameters §	Influenza	Risk of Bias
Barnett et al.	2006	M	N	0–1	ICD-9, ICD-10 ¢	Y	Y	Y	Y	N	Low
Talbott et al.	2014	M	Y	0	ICD-9	Y	Y	Y	Y	N	Low
Guo et al.	2008	S	Y	0	ICD-10	Y	Y	Y	Y	N	Low
Chiu et al.	2013	S	Y	0	ICD-9	Y	Y	Y	Y	N	Low
Tsai et al.	2009	S	Y	0	ICD-9	Y	Y	Y	Y	N	Low
Bunch et al.	2011	S	Y	0	NR ♯	Y	Y	Y	Y	N	High
Zhao et al.	2014	S	Y	0	Electrocardiogram Criteria	Y	Y	Y	Y	N	Low
Santos et al.	2008	S	Y	0	ICD-10	Y	Y	Y	Y	N	Low
Ueda et al.	2009	M	Y	0	ICD-10	Y	Y	Y	Y	N	Low
Poloniecki et al.	1997	S	Y	1	ICD-9	Y	Y	Y	Y	N	Low
Hoek et al.	2011	M	Y	0-6	ICD-9	Y	Y	Y	Y	N	Low
Burnett et al.	1999	S	Y	1	ICD-9	Y	Y	Y	Y	N	Low
Halonen et al.	2009	S	Y	0	ICD-10	Y	Y	Y	Y	Y	Low
Koken et al.	2003	S	Y	0	ICD-9	Y	N	N	Y	N	High
Linn et al.	2000	S	Y	0	APR-DRG ¶	Y	Y	Y	Y	N	Low
Stieb et al.	2009	M	Y	0	ICD-9, ICD-10	Y	Y	Y	Y	N	Low
Haley et al.	2009	S	Y	0	ICD-9	Y	Y	Y	Y	N	Low
Chiusolo et al.	2011	M	Y	0-5	ICD-9	Y	Y	Y	Y	Y	Low
Peel et al.	2007	S	N	0-2	ICD-9	Y	Y	Y	Y	N	Low
Colais et al.	2012	M	Y	0	ICD-9	Y	Y	Y	Y	Y	Low
Chang et al.	2015	S	N	0-2	ICD-9	Y	Y	Y	Y	N	Low
Milojevic et al.	2014	M	N	0-4	ICD-10	Y	Y	Y	Y	N	Low
Tolbert et al.	2000	S	Y	0	ICD-9	Y	Y	Y	Y	N	Low
Goldberg et al.	2013	S	Y	0	ICD-9	Y	Y	Y	Y	N	Low
Lippmann et al.	2000	S	Y	0	ICD-9	Y	Y	Y	Y	Y	Low

* Shortest Lag: The shortest lag day presented in study to analyze the association between air pollution and arrhythmia. § Meteorological Parameters: Refers to temperature and humidity in our study. ¶ APR-DRG: All-Patient-Refined Diagnosis-Related Group. ¢ ICD: International Classification of Diseases. ♯ NR: Not Reported. Y: Yes. N: No. S: Single City. M: Multi City.

Table S4. Daily concentrations of air pollutants by geographical location.

Air Pollutants	Median #	First Quartile	Third Quartile
$PM_{2.5}$ (μg/m^3) ¶			
Europe	9.750	6.250	13.350
North America	9.673	6.382	14.103
Asia	30.153	19.224	41.453
PM_{10} (μg/m^3)			
Europe	30.737	21.530	48.222
North America	32.587	23.356	42.484
Asia	81.313	54.407	116.867
CO (ppm)			
Europe	0.475	0.375	0.801
North America	1.248	0.843	1.724
Asia	0.940	0.700	1.190
O_3 (ppb)			
Europe	21.133	13.139	32.728
North America	25.335	17.353	34.134
Asia	22.520	16.230	28.660
NO_2 (ppb)			
Europe	20.533	14.320	28.138
North America	29.361	22.023	36.932
Asia	25.785	20.223	32.263
SO_2 (ppb)			
Europe	4.038	2.333	9.257
North America	6.974	3.164	11.153
Asia	7.475	5.043	11.143

¶ PM: Particulate Matter. # Median, first quartile and third quartile pollutant concentration derived from the average daily pollutant concentrations reported per study.

© 2016 by the authors; licensee MDPI, Basel, Switzerland. This article is an open access article distributed under the terms and conditions of the Creative Commons by Attribution (CC-BY) license (http://creativecommons.org/licenses/by/4.0/).
